# Prediction of Suitable Regions for *Danxiaorchis yangii* Combined with Pollinators Based on the SDM Model

**DOI:** 10.3390/plants13213101

**Published:** 2024-11-03

**Authors:** Xuedie Liu, Can Gao, Guo Yang, Boyun Yang

**Affiliations:** Life of Sciences, Nanchang University, Nanchang 330031, China; liuxuedie1995@126.com (X.L.); gc1234560318@163.com (C.G.); 405600220016@email.ncu.edu.cn (G.Y.)

**Keywords:** *Danxiaorchis yangii*, *Dufourea* spp., *Lysimachia alfredii*, suitable region

## Abstract

*Danxiaorchis yangii*, a newly discovered fully mycoheterotrophic orchid. It relies on *Lysimachia alfredii* and *Dufourea* spp. for pollination, and environmental factors closely influence the growth and distribution of these pollinators, which in turn directly affects the growth and reproduction of *D. yangii*. Climate change threatens the suitable habitats for these three species, emphasizing the need to understand *D. yangii*’s response. This study comprehensively utilized the field distribution of *D. yangii* and related climatic data, along with future climate predictions from global models, to predict the climate suitability areas of *D. yangii* under two greenhouse gas emission scenarios (SSP245 and SSP370) using species distribution models (SDMs), which encompassed a random forest (RF) model. Additionally, we selected the optimal ensemble model (OEM) for *Dufourea* spp. and applied generalized boosted models (GBMs) and RF for *L*. *alfredii* in our predictions. The study found that precipitation of the driest quarter plays a pivotal role in determining the distribution of *D. yangii*, with an optimal range of 159 to 730 mm being most conducive to its growth. Comparative analysis further indicated that precipitation exerts a greater influence on *D. yangii* than temperature. Historically, *D. yangii* has been predominantly distributed across Jiangxi, Hunan, Zhejiang, and the Guangxi Zhuang Autonomous Region, with Jiangxi Province containing the largest area of highly suitable habitat, and this distribution largely overlaps with the suitable regions of its pollinators.

## 1. Introduction

Climate change is an ongoing and cyclical phenomenon influenced by Milankovitch cycles, atmospheric disturbances from volcanic activity, and significant shifts in photosynthetic biomass throughout Earth’s history [1]. In the face of rapidly changing climate conditions, species can respond in various ways: through acclimation or adaptation, migration and range modification, or extinction [2]. For species with discontinuous or highly fragmented habitats, particularly rare species, the risks of migration challenges and extinction are heightened. Species distribution models (SDMs), also known as environmental or ecological niche modeling, integrate species locality data and other biological diversity attributes with environmental predictors, employing statistical and machine learning techniques to produce empirical descriptions and spatial predictions of species–environment relationships [3,4]. Currently, SDMs are widely utilized to analyze the impact of climate change on potential species distributions, with model simulations predicting changes in suitable habitats across different climate scenarios [5]. It is widely recognized that species distribution and abundance are not only determined by abiotic factors but also by other ones [6]. Using species distribution models (SDMs) without considering biotic interactions, such as plant–pollinator relationships in *Ophrys argolica* and *O. delphinensis* [7], or trophic interaction in *Nucifraga caryocatac* [8], may overestimate suitable areas for species that rely on others. Therefore, biotic interactions are increasingly incorporated into SDMs or combined with their outcomes.

Members of Orchidaceae are long-lived perennials, with generation times exceeding 100 years in some cases, and are globally distributed [9]. Orchids produce large numbers of wind-dispersed “dust” seeds per fruit [10] that lack endosperm and constitute little more than air-filled casings around the embryo. Orchid seeds exhibit extremely low germination rates because only a small portion of the large number of seeds will land on a suitable substrate and eventually germinate, and throughout their life cycle, orchid plants depend not only on the induction and symbiosis of specific fungal groups but also on stringent environmental conditions to support their normal growth [11,12]. In addition to these challenges, numerous species within this family are declining globally due to habitat loss, climate change, and shifts in species distributions pollinated by specific pollinators. Consequently, their distribution is influenced not only by climate and site characteristics (e.g., geology) but also by interactions with biotic factors (e.g., mycorrhizal fungi, pollinators) [13].

*Danxiaorchis* (Calypsoinae, Epidendreae, Epidendroideae), a recently described fully mycoheterotrophic orchid genus, was characterized by a distinct Y-shaped callus in its labellum [14]. Species of the *Danxiaorchis* genus lack both leaves and roots, making them unable to obtain the necessary nutrients for growth through photosynthesis. Instead, they sustain themselves by drawing nutrients from fungi in their surrounding environment, with which they have a mutually beneficial and interdependent relationship. Due to their extreme reliance on these fungi, they find it very difficult to survive when transplanted to other locations. Only three species of *Danxiaorchis* have been described so far: *Danxiaorchis singchiana* [15], *Danxiaorchis yangii* [16], and *Danxiaorchis mangdangshanensis* [17]. *D. yangii*, a holomycotrophic new species from Jinggang Mountain National Nature Reserve, western Jiangxi, eastern China. In previous work, we found that *D. yangii* employs Batesian mimicry, imitating the floral morphology of *Lysimachia alfredii* to attract *Dufourea* spp. for pollination [18]. Our field investigations revealed that *D. yangii* prefers steep slopes with high canopy closure and cool, moist conditions, often forming small clusters scattered across multiple areas of the same mountain range. However, the specific environmental factors and biotic factors that influence the distribution of *D. yangii* remain unknown.

Based on this background, the aim of this study was to explore how *D. yangii*’s potential distribution was affected by plant–pollinator interactions under current and future climatic conditions, utilizing high-resolution environmental data at 30 arc-seconds and the flexible SDMs package Biomod2, in conjunction with ArcGIS software (Version 10.8). The major objectives were the following: (1) to predict the current and future distribution of the potential suitable growth areas of *D. yangii*; (2) to explore the impact of dominant environmental factors and its unique pollinators on the distribution of *D. yangii*, providing a scientific basis for ecological protection and resource utilization.

## 2. Materials and Methods

### 2.1. Collection of Distribution Points

*D. yangii* was first discovered and described in 2017 from the Jinggang Mountain National Nature Reserve, Jiangxi Province, China [16], and was also found in Hunan Province. *D. yangii* typically grows at an altitude of 300–750 m on the edges of subtropical evergreen broadleaf forests and in mixed forests of shrubs and bamboo. Currently, there are no other recorded distributions of *D. yangii*. All distributions used in this study were provided by our lab investigations. Additionally, we collected the natural distribution of *Dufourea* spp. and *L. alfredii* to analyze the relationship between *D. yangi* and its pollinators. The distribution data for *Dufourea* spp. and *L. alfredii* were primarily sourced from the Chinese Virtual Herbarium (accessed on 6 July 2024; http://www.cvh.ac.cn/), National Specimen Information Infrastructure Teaching Specimen Resource Sharing Platform (accessed on 6 July 2024; http://mnh.scu.edu.cn), Chinese Natural Specimen Resource Platform (accessed on 6 July 2024; http://www.cfh.ac.cn/), National Specimen Information Infrastructure (accessed on 6 July 2024; http://www.nsii.org.cn/), Chinese Plant Photo Bank (accessed on 6 July 2024; http://ppbc.iplant.cn/), Global Biodiversity Information Facility (https://www.GBIF.org (accessed on 5 July 2024) GBIF Occurrence Download https://doi.org/10.15468/dl.cuz38w; GBIF, https://www.GBIF.org (accessed on 5 July 2024) GBIF Occurrence Download https://doi.org/10.15468/dl.u9vfdc) [19], as well as relevant books such as the Flora of China, Flora of Yunnan, and other related research literature [20]. Only distribution points recorded after 1970 were retained, and for records without coordinates, latitude and longitude data were assigned via Google Earth (accessed on 7 July 2024; http://ditu.google.cn/) [21]. Misidentified specimen points were verified and removed, and duplicate points too close together were eliminated using the dist_mat command in R, retaining only one point per 30 arc-seconds (approximately 1 km) grid [22]. Finally, we obtained 10 distribution points for *D. yangii*, 43 for *Dufourea* spp., and 217 for *L. alfredii* in China.

### 2.2. Environmental Data Preprocessing

In our study, climate is considered a key variable to explore the potential impacts of climate change on *D. yangii* from historical (near-current) times to the end of the century. We used 19 bioclimatic variables from the WorldClim database (http://www.worldclim.org/, accessed on 1 August 2024) as the main factors for SDM to predict the distribution patterns of *D. yangii*, *Dufourea* spp., and *L. alfredii* [23]. Since elevation differences at the same latitude affect temperature and precipitation, influencing the distribution of plants and their pollinators, we also used elevation data from WorldClim to examine the relationship between elevation and distribution patterns [24]. Before use, we screened the environmental variables by importing the 19 bioclimatic variables and species distribution data into ArcGIS V10.4. Climate data corresponding to the distribution points were extracted, and correlation analysis was conducted in R v4.4.1 [25]. Variables with a correlation coefficient r > |0.8| were excluded [26]. The remaining climatic factors were retained for further modeling.

Historical (1970–2000) and future climate data were obtained from the WorldClim2.1 dataset based on CMIP6, with projections for four periods: 2021–2040 (2030s), 2041–2060 (2050s), 2061–2080 (2070s), and 2081–2100 (2090s) under two climate scenarios, SSP245 and SSP370. The WorldClim2.1 dataset includes up to 25 global climate models; for this prediction, we selected the EC-Earth3-Veg model (the European Community Earth-system model version 3.3 for vegetation) developed by the European Centre for Medium-Range Weather Forecasts, due to its superior performance in predicting precipitation in China [27,28] (Table 1). All environmental variables were masked and extracted for the Chinese region using ArcGIS 10.4, with a uniform resolution of 30 arc-seconds and the WGS1984 coordinate system, saved as .TIF files for use in Biomod2 modeling.

### 2.3. Construction and Validation of the SDM

Biomod2, a modeling platform based on R software, was developed in 2003 [29,30]. It is an R package that includes the following species distribution modeling algorithms: generalized linear models (GLM), generalized boosted models (GBM), generalized additive models (GAM), classification tree analysis (CTA), artificial neural networks (ANN), surface range envelope (SRE), flexible discriminant analysis (FDA), multivariate adaptive regression splines (MARS), random forest (RF), and maximum entropy models (MaxEnt), of which GBM and RF were finally used in our study.

Following the ODMAP (overview, data, model, assessment, and prediction) protocol, we sequentially constructed the ecological niche model workflow from five parts: overview, data, model, assessment, and prediction [31]. In our study, the ENMeval package [29] was used to optimize two key parameters of the MaxEnt model at the species level in order to minimize overfitting and sampling bias while improving predictive accuracy. Subsequently, ten individual models were run in the Biomod2 package, and models meeting the selection criteria were re-integrated to form ensemble models. The most suitable model for the target species was then selected for subsequent suitable region simulation. It is necessary to optimize two key MaxEnt parameters using the ENMeval 2.0 package [32]: regularization multiplier (RM) and feature combination (FC). RM values range from 0.5 to 4, in increments of 0.5. FCs are combinations of five features: linear (L), quadratic (Q), hinge (H), product (P), and threshold (T), resulting in combinations such as L, LQ, LQH, H, LQHP, and LQHPT. By cross-pairing the 8 RMs and 6 FCs, 48 parameter combinations are formed. Subsequently, the ENMeval test is performed, and the parameter combination with the lowest AICc increment (delta.AICc), typically zero, and a high AUC value (AUC > 0.9) is selected to optimize MaxEnt [33]. In addition to the parameter tuning required for MaxEnt, other single models such as GLM, GAM, GBM, CTA, ANN, SRE, FDA, MARS, and RF use default parameters. Using Biomod4.1-2, optimal ensemble models for *D. yangii*, *Dufourea* spp., and *L. alfredii* are constructed through the following main steps: First, single models and the full ensemble model (FEM) are used to model the target species, and the evaluation metrics of the models are output. Next, high-accuracy single models are selected based on these metrics and recombined to form the optimal ensemble model (OEM), which is then evaluated. Finally, by comparing the evaluation metrics of each model, the one with the best predictive ability is selected to simulate suitable regions under both historical and future scenarios [21].

The model evaluation metrics used include true skill statistic (TSS) and the area under the ROC curve (AUC). TSS values range from [−1, 1], with values closer to 1 indicating higher model accuracy [34]. An AUC > 0.95 signifies an excellent model [35]. These metrics are employed to select the best comprehensive model. For *Dufourea* spp., since the evaluation results did not meet the criteria for excellence, the threshold was adjusted to AUC > 0.8 to select satisfactory models [35]. All operations were performed in R, with 100 random background points generated for *D. yangii* and 500 for *Dufourea* spp. and *L. alfredii*. The distribution data were split, with 75% used for training and 25% for testing, and the model was run five times [21,36].

### 2.4. Visualization Analysis

In constructing the models, weights were assigned according to TSS values, with higher TSS values corresponding to higher weights for the respective model [37]. The “get_variables_importance” command in R was used to generate box plots, illustrating the importance of each variable in the model [29,30]. The “bm_PlotResponseCurves” command was employed to draw bivariate response curves, setting environmental variables to median values to assess the species’ response under moderate environmental conditions. The projection results for each period, simulated by the optimal ensemble model (OEM), were visualized in ArcGIS 10.4 using the reclassification tool to divide the suitability into four categories: no suitability (grid value = 0), low suitability (0 < grid value ≤ 0.4), medium suitability (0.4 < grid value ≤ 0.6), and high suitability (0.6 < grid value ≤ 1) [20]. The binary suitability threshold was determined using the maximized TSS principle, and binary distribution maps were created accordingly. Using the SDM toolbox v2.5 plugin [38], regions of expansion, contraction, stability, and centroid migration trajectories for the species under future climate scenarios were generated.

To avoid the confounding effects of multiple environmental factors on the climatic impact, we used only temperature and precipitation-related variables in the modeling process. However, in the actual environment, we found that topographical and biological factors also significantly affect the life history of *D. yangii*. Therefore, we examined in detail the relationships between key environmental factors, including elevation, slope, aspect, pollinators, and their foraging plants, in different levels of suitable regions for *D. yangii* under historical climates. Key environmental factors were extracted from the binary distribution maps to analyze the changes in environmental variables within suitable regions and their relationships [39,40].

## 3. Results

### 3.1. Model Optimization and Selection

**Figure 1:**
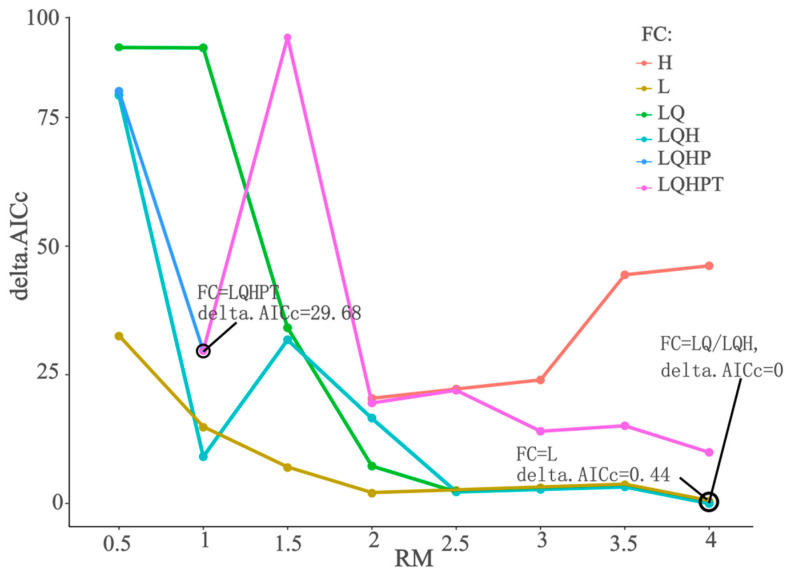
Model accuracy for *D. yangii* under 48 parameter combinations.

Before modeling *D. yangii*, *Dufourea* spp., and *L. alfredii*, MaxEnt’s key parameters were optimized. The optimal parameter combinations for RM and FC were [4, LQ], [1, LQHPT], and [1, LQHP], respectively. For *Dufourea* spp. modeling, the default parameters were already the optimal combination. Taking *D. yangii* as an example (Figure 1), the default parameters resulted in a delta.AICc of 29.68, whereas the optimized parameter combination achieved a delta.AICc of 0, indicating no model increment and the best performance. Notably, *D. yangii* also had a delta.AICc of 0 with the [4, LQH] combination, but the [4, LQ] model was simpler. In selecting the parameter combination for modeling, we found that the optimized MaxEnt surpassed previous evaluation metrics, prompting us to choose the best-performing model for further simulations. In the *D. yangii* simulation, the OEM performed well but was slightly less effective than the RF model: RF had a TSS value of 0.9968, which was 0.080 higher than OEM, and an AUC value of 0.9984, exceeding OEM by 0.024. Given RF’s simplicity, we opted to use it directly for *D. yangii* predictions across various scenarios. For *Dufourea* spp. and *L. alfredii*, we selected the OEM based on GLM, GBM, MARS, RF, MaxEnt.2, and GBM, RF, respectively, for predictions.

### 3.2. Analysis of Suitable Regions Under Historical Climate

**Figure 2:**
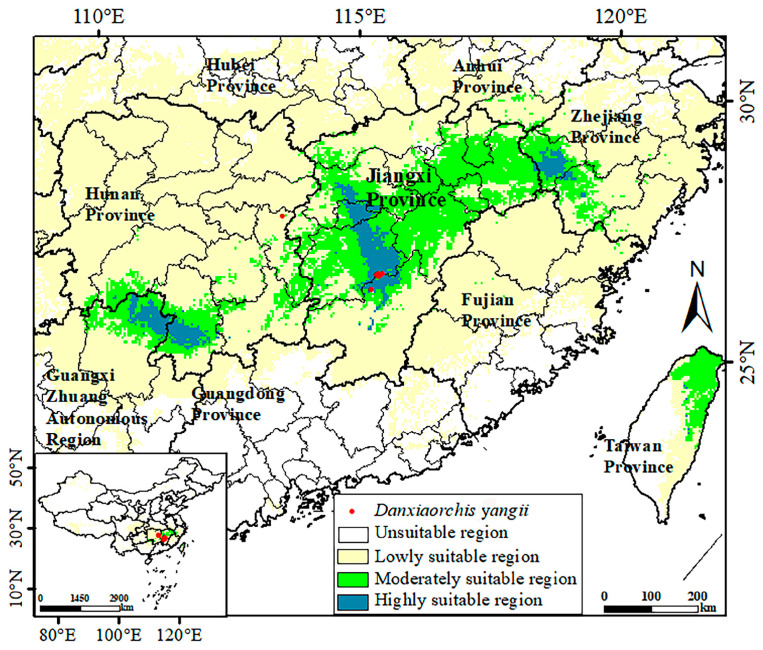
Potential distribution and actual distribution points of *D. yangii* under the historical environmental condition.

The prediction results show that *D. yangii* historically thrived in a broad area within Jiangnan, China, with a suitable habitat covering approximately 37.26 × 10^4^ km^2^. This included the entire Jiangxi Province, parts of southeastern Hunan, southern Zhejiang Province, northeastern Guangxi Zhuang Autonomous Region, and even northeastern Taiwan, all exhibiting favorable climatic conditions for *D. yangii* growth (Figure 2). Among these, 38.3% of this region was classified as highly suitable for *D. yangii*, predominantly located in three areas: one in the northeastern part of Guilin and central Yongzhou, another comprising central Yichun, extending across central and eastern Xinyu, Ji’an, and northern Ganzhou—considered the main historical distribution area of *D. yangii*. The last area was covered in central and southern Quzhou and northwestern Lishui City of Hunan Province. The moderately suitable regions (48.3%) formed a belt connecting these highly suitable zones, with notable concentrations in Zhejiang, Jiangxi, Hunan, and Guangxi provinces. Additionally, these moderately suitable regions also extended to the northern and eastern coastal regions of Taiwan. Lastly, 13.4% of the region was deemed lowly suitable, forming an expansion outward from the moderately suitable zones.

The map highlights Jiangxi Province as the key area for protecting *D. yangii*, given that the climate in most parts of the province is highly suitable for its survival. This area also contains the original habitat of *D. yangii*, making it essential for conservation efforts. For future reintroduction efforts, priority can be given to the highly suitable regions within Jiangxi, especially in areas like central Yichun, Ji’an, eastern Xinyu, and northern Ganzhou. Additionally, the other two highly suitable regions—northeastern Guilin and central Yongzhou, and central and southern Quzhou and northwestern Lishui—should also be considered to expand the protection and potential recovery of *D. yangii* in the wild.

### 3.3. Environmental Factors Analysis

**Figure 3:**
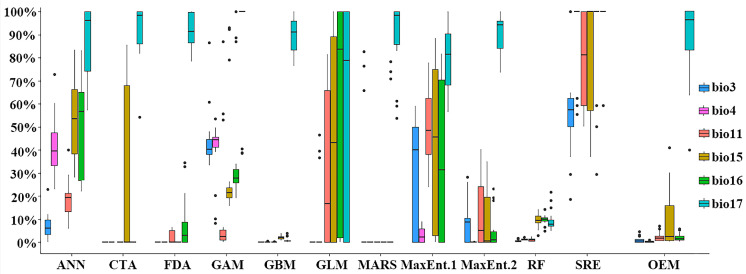
Importance of environmental factors evaluated by each model.

We found that different models exhibited varying sensitivity to the climatic factors affecting the distribution of *D. yangii*. However, it is evident that the precipitation of the driest quarter (bio17) was the most prominent factor across all models, followed by precipitation seasonality (bio15) and the precipitation of the wettest quarter (bio16) (Figure 3). In our modeling, we selected three environmental variables, each related to temperature and precipitation. All models showed a preference for precipitation-related factors, which had a significant impact on the distribution of *D. yangii*. From a topographical perspective, the moderately and highly suitable regions for *D. yangii* are surrounded by the Xuefeng, Nanling, and Wuyi mountain ranges. The three main highly suitable regions are located between the southern side of the Xuefeng Mountains and the northern side of the Nanling Mountains, the northern side of central Wuyi Mountains, and the northern side of the northern Wuyi Mountains. In the context of gradual global warming, the suitable region for *D. yangii* is expected to expand rather than decrease, likely due to the strong topographical heterogeneity of the surrounding mountains. This heterogeneity causes the southeast and southwest monsoons to bring precipitation that remains in these areas, forming microclimates that provide refuges for *D. yangii* [38,39].

**Figure 4:**
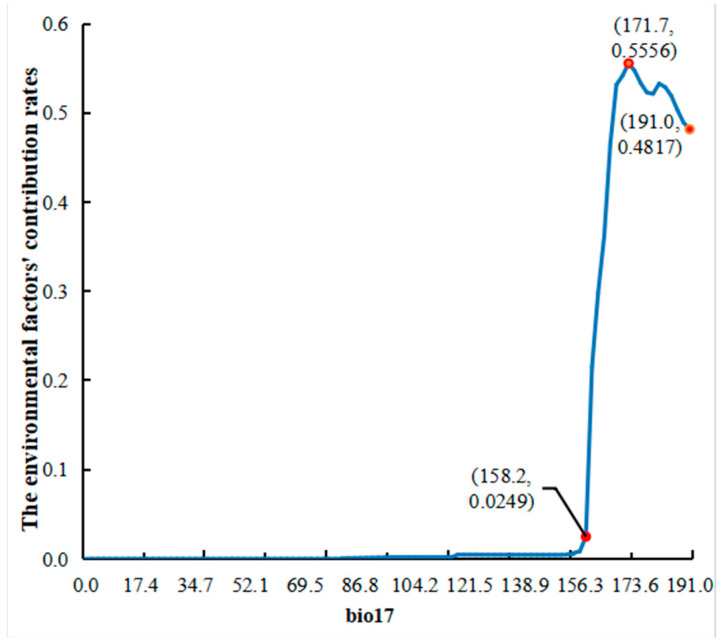
Response curves for important environmental factors.

The response curves of environmental factors (Figure 4) provide insights into the relationship between environmental variables and habitat suitability for the target species. When the contribution rate of an environmental factor exceeds 0.5, it indicates that the corresponding variable range is most favorable for the target species’ survival [37]. The response curve for bio17 (precipitation of the driest quarter), which has the highest contribution rate, shows a sharp increase in its contribution rate to 0.0249 when the precipitation of the driest quarter exceeds 158.2 mm, peaking at 171.7 mm, and then fluctuating with a decreasing trend, reaching 0.4817 at 191.0 mm.

We extracted bio17 for each suitable region level and obtained the following results: in China, the precipitation of the driest quarter ranges from [0, 764]. Specifically, bio17 in the lowly suitable region ranges from [0, 397], in the moderately suitable region from [159, 764], and in the highly suitable region from [159, 320]. This is consistent with the results of the environmental response curve mentioned above, indicating that a precipitation of the driest quarter exceeding 159 is more favorable for the survival of *D. yangii*. Upon extracting elevation data, we found that the elevation in the lowly suitable region for *D. yangii* ranges from [−153, 7200], in the moderately suitable region from [4, 3510], and in the highly suitable region from [33, 2265]. The variation in elevation across different suitable region levels is maximal, with *D. yangii* primarily concentrated below 3500 m and thriving best below 2300 m. Additionally, we analyzed aspect and slope data and found that *D. yangii* prefers to grow on steeper slopes but shows no particular preference for aspect.

**Figure 5:**
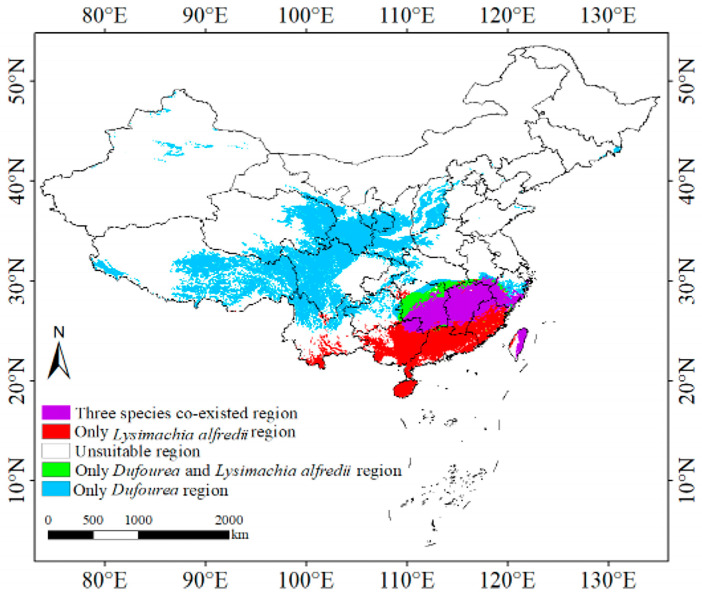
Overlap map of the distribution of the three species determined by the TSS maximization principle.

**Figure 6:**
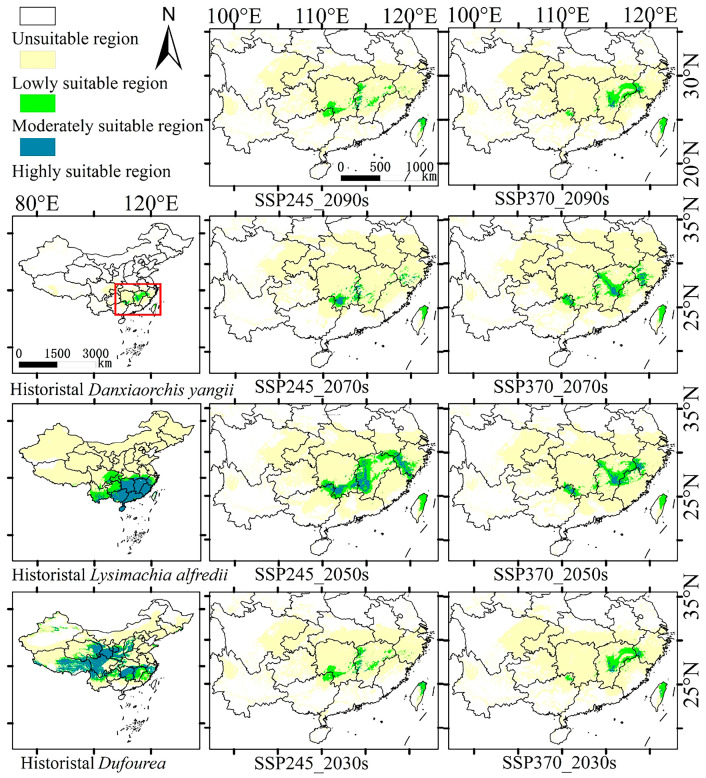
Prediction of potential suitable region.

By predicting the suitable regions for *L. alfredii* and *Dufourea* spp. under historical climate conditions (Figure 5 and Figure 6), we found that the suitable region for *D. yangii* largely overlaps with the suitable regions of these two species. This suggests that the suitable region of *D. yangii* falls within the range of its pollinators and the plants on which these pollinators feed. Under historical climate conditions, the distribution of the pollinator *Dufourea* spp. was the most extensive, covering central southwestern China, southeastern northwest, and southern central and eastern China. *L. alfredii* was primarily distributed across southern China, southern central China, southeastern China, and southwestern China. In contrast, *D. yangii* was mainly concentrated in southern central and southeastern China. When the suitable regions of the three species were overlaid (Figure 5), it became evident that *D. yangii* is located at the intersection of the ranges of *L. alfredii* and *Dufourea* spp. Given the pollination requirements of *D. yangii* [18], this overlap suggests that the availability of its pollinators might be a key factor limiting its distribution range.

### 3.4. Changes in Suitable Regions Under Future Climates

As shown in Figure 6, the future climate is projected to be more suitable for the survival of *D. yangii* compared to historical climates. The total area of suitable regions is expected to increase, reaching its maximum in the 2050s under the SSP245 pathway. Under the SSP370 pathway, both moderately and highly suitable regions in the 2050s will be maximized across all future scenarios, second only to the SSP245 pathway for the same period. Regardless of how the climate changes, the overall trend shows that the total suitable regions for *D. yangii* will increase, peaking in the 2050s, before gradually decreasing. Among the pathways, the SSP245 pathway is the most favorable for *D. yangii*, as it will lead to the highly suitable regions gradually converging and forming a large, continuous area in the central and northern parts of Jiangxi Province. Comparing this to the distribution patterns under historical climates, it is clear that the central part of Jiangxi Province consistently exhibits higher suitability (Figure 6).

**Figure 7:**
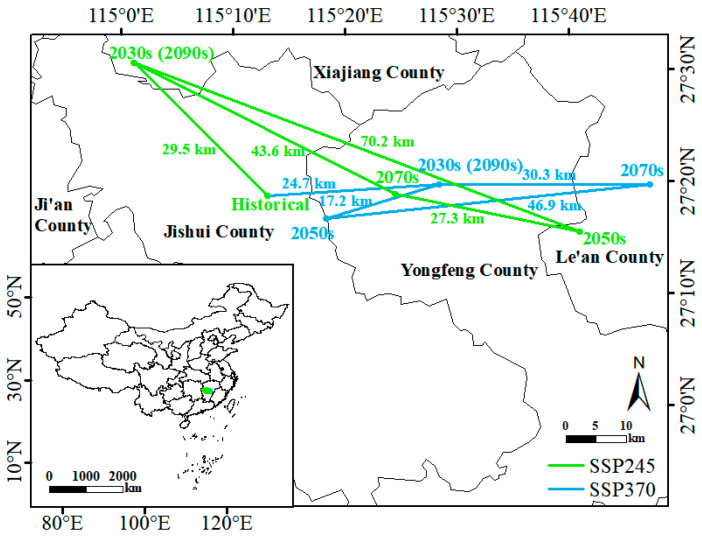
Shifts of centroids of *D. yangii* under different climate scenarios.

Furthermore, regardless of climate changes, the centroid of *D. yangii*’s suitable region will generally remain near Ji’an City and Fuzhou City in Jiangxi Province. Under historical climates, the centroid is located in Jishui County. By the 2030s, under SSP245 and SSP370, the centroid will shift 29.5 km to the northwest and 24.7 km to the east, respectively. Despite variations in the centroid during the 2050s and 2070s, it will eventually return to its 2030s position by the 2090s (Figure 7).

**Figure 8:**
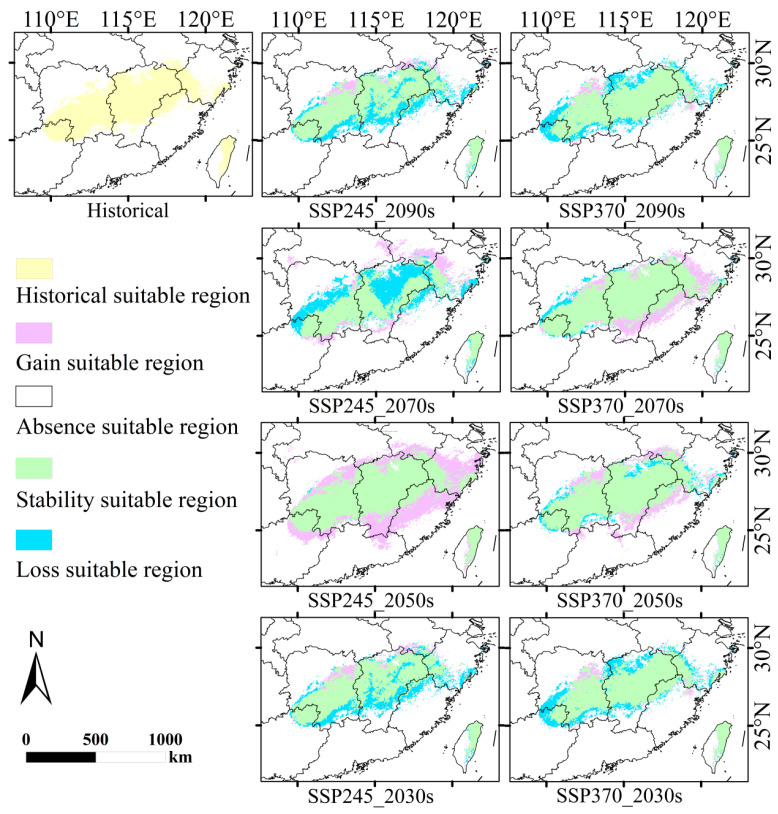
Spatial distribution pattern changes in *D. yangii* under different climate scenarios.

Compared to the suitable regions under historical climates, the reduction in suitable regions for *D. yangii* in the future will primarily occur at the edges of the lowly suitable regions, with minimal impact on the moderately and highly suitable regions (Figure 8). Under the SSP245 pathway, there will be a significant increase in the total area of suitable regions by the 2050s, characterized by an expansion in all directions. Moreover, the overall change in suitable regions compared to historical climates will be relatively minor, with only slight losses in the southwestern part of the suitable regions and small gains at the eastern and northern edges. This suggests that the core areas for *D. yangii*, particularly the moderately and highly suitable regions, will remain largely intact and may even expand under future climate conditions, while only marginal areas at the periphery of its range will experience some reduction.

## 4. Discussion

### 4.1. Prospective Changes in D. yangii’s Suitable Habitat

Rapid climate change is currently outpacing the ability of many plant species to adapt, creating mismatches between the climatic conditions and the preferences of species within a community [40]. Environmental factors play a crucial role in driving species growth [34], and with rising global temperatures and shifting precipitation patterns, it is vital to simulate the potential distribution of plants for effective ecological and practical management of species and forest ecosystems. Identifying the environmental factors that shape and maintain the geographic distribution of species is essential from both evolutionary and ecological perspectives [41]. Numerous studies have shown that temperature and precipitation are the most direct and critical factors affecting vegetation [42]. Precipitation is the most significant climatic variable shaping the environmental conditions of a region, influencing hydrological hazards like floods and droughts as well as determining species distribution and growth conditions [43]. Mithilasri et al. (2024) revealed that among bioclimatic variables, the annual temperature range (Bio 7) contributes the most (31.44%) to the distribution of *Bulbophyllum acutiflorum*, followed by precipitation seasonality (Bio 15) at 25.44%, indicating that this species may tolerate varying temperatures and prefer habitats with distinct wet and dry seasons [44]. In this study, the key environmental factors influencing the growth and distribution of *D. yangii* were identified as the precipitation of the driest quarter (Bio17), precipitation seasonality (Bio15), and precipitation of the wettest quarter (Bio16) (Table 1), suggesting that *D. yangii* prefers habitats with sufficient precipitations. In future climate scenarios, the suitable habitat for *D. yangii* will continue to expand until the 2070s and then gradually decline. However, the central region of Jiangxi Province (Ji’an City and Fuzhou City) will remain a highly suitable habitat and should be prioritized for conservation. This is likely due to the fact that the preferred habitat for *D. yangii* is primarily concentrated in warm and humid climates, fertile soils, and moderate light conditions, which closely align with the climatic and topographical conditions of Ji’an and Fuzhou in Jiangxi Province. Mountainous regions of suitable habitat with high topographic diversity may provide refuge by buffering disturbances due to the presence of microhabitats.

### 4.2. Strengths and Limitations of the Study

While we used Biomod2 for the first time to model the potential habitat of *D. yangii* in China under present and future scenarios (SSP245 and SSP370) for the 2030s, 2050s, 2070s, and 2090s, there are still some limitations. (1) The sample data of *D. yangii* only covers Jiangxi Province. The limited and dispersed distribution points increased uncertainty in our model, potentially leading to discrepancies from the actual suitable areas. The resolution of the environmental data used may not adequately capture microhabitat variations that are crucial for the survival of *D. yangii*, possibly resulting in an oversimplified prediction. (2) In our study, the environmental variables that may significantly influence the distribution of *D. yangii* were divided into two categories: one consisting of temperature and precipitation factors for modeling purposes, and the other comprising topographical and pollination-related biotic factors, which were used for a comprehensive analysis of the modeling results. This method of categorizing and analyzing environmental factors not only helps to avoid the issue of multiple environmental variables intertwining and overshadowing the role of climatic factors but also allows for a clearer understanding of the importance of each type of environmental variable and the specific environmental requirements for the distribution of *D. yangii*. The biotic interactions, ultraviolet irradiation, and plateau meteorology were not included in this study because of a lack of accurate data on these variables. Limitations in spatial data, and the assumption that species could migrate to climate-friendly areas under climate change, have led to uncertainty in species distribution projections [45]. (3) Previous studies have extensively explored temperate European orchids, analyzing the biotic and abiotic factors that influence their presence, abundance, and distribution [46,47,48]. Orchids form mycorrhizal symbioses with fungi in natural habitats, affecting their seed germination, protocorm growth, and nutritional acquisition [49]. Fungal symbionts are essential as they provide carbon and minerals to the dust-like, reservoir-less orchid seeds during early development [50]. The distribution of orchids is intricately linked to their reliance on specific mycorrhizal fungi for nutrient acquisition, which poses several limitations. Factors such as soil type, moisture levels, and overall ecosystem health determine the presence and abundance of these mycorrhizal fungi. For instance, Pica et al. (2024) examined the current and potential future distribution of three forest orchids (*Cephalanthera*, *Epipactis*, and *Limodorum*) in a protected area of the Northern Apennines, using habitat suitability models based on presence-only data and the MaxEnt model, finding that precipitation-related variables (BIO04, BIO11, BIO14, BIO15, BIO18) and soil variables (the bulk density of the fine earth fraction (SOIL1), the volumetric fraction of coarse fragments (SOIL3), and the proportion of clay particles in the fine earth fraction (SOIL4)) significantly influenced their distribution [51]. Additionally, Smallwood et al. (2022) found that edaphic conditions significantly influence the distribution of six *Cypripedium* spp. due to their role in supporting obligate mycorrhizal symbionts, suggesting that range modifications of terrestrial orchids in response to climate change may only be feasible if multiple co-occurring species respond similarly [2].

Consequently, any changes in the environment—such as climate change, habitat destruction, or pollution—can impact fungal populations, thereby restricting the growth and reproductive success of orchids. Additionally, the specificity of some orchids to particular fungal partners means that the loss of these fungi could lead to local extinctions of these orchids. Furthermore, the geographic distribution of orchids and their associated fungi is often limited by environmental factors such as altitude, temperature, and precipitation, which can create microhabitats suitable for one but not the other. Therefore, we suggest that future studies should collect more detailed data by increasing field investigations and supply missing impact factors, such as fungal distribution data, to enhance the precision and reliability of the predictive outcomes.

## 5. Conclusions

In this study, we explored the response of *D. yangii* to climate change in China by predicting the potential distribution of suitable niches using the Biomod2 model based on distribution records. Our findings reveal several key points: (1) The main environmental variables constraining the distribution include the precipitation of the driest quarter (Bio17), precipitation seasonality (Bio15), and precipitation of the wettest quarter (Bio16); (2) currently, suitable niches for *D. yangii* are primally located in Jiangxi Province, with highly suitable areas concentrated in Ji’an City and Fuzhou City. Notably, under historical climate conditions, the distribution of *D. yangii* largely overlaps with the suitable regions of its pollinators (*L. alfredii* and *Dufourea* spp.); (3) under future climate scenarios, the potential suitable regions are expected to expand significantly before the 2070s, particularly with a notable increase in highly suitable areas in central Jiangxi Province. This study is the first to predict the suitable habitat distribution of *D. yangii* based on environmental factors in conjunction with its pollinators under current and future climate scenarios, and we hope our findings will provide new insights for its conservation.

## Figures and Tables

**Table 1:** Environmental variables (variables in bold are used for modeling *D. yangii*).

Environmental Variables (Code)	Units
Mean annual air temperature (bio1)	°C
Mean diurnal range (bio2)	°C
Isothermality (bio3 = (bio1/bio7) × 100)	-
Variation of temperature seasonlity (bio4)	C of V
Maximum temperature of warmest month (bio5)	°C
Minimum temperature of coldest month (bio6)	°C
Temperature annual range (bio7)	°C
Mean temperature of wettest quarter (bio8)	°C
Mean temperature of driest quarter (bio9)	°C
Mean temperature of warmest quarter (bio10)	°C
Mean temperature of coldest quarter (bio11)	°C
Mean annual precipitation (bio12)	mm
Precipitation of wettest month (bio13)	mm
Precipitation of the driest month (bio14)	mm
Variation of precipitation seasonlity (bio15)	C of V
Precipitation of wettest quarter (bio16)	mm
Precipitation of driest quarter (bio17)	mm
Precipitation of warmest quarter (bio18)	mm
Precipitation of coldest quarter (bio19)	mm

## Data Availability

Data are contained within the article.
